# Supplementary material for: Gender Differences Associated with the Prognostic Value of BPIFB4 in COVID-19 Patients: A Single-Center Preliminary Study
Source: J Pers Med. 2022 Jun 28;12(7):1058. doi: 10.3390/jpm12071058 (PMC9319362; doi:10.3390/jpm12071058)
Supplement: Supplementary file 1 [file jpm-12-01058-s001.zip › jpm-1771345-supplementary.pdf]

Gender Differences Associated with the Prognostic Value of BPIFB4 in COVID-19 Patients: A Single-Center Preliminary Study

Supplementary Materials

jpm-1771345.supplementary.word.

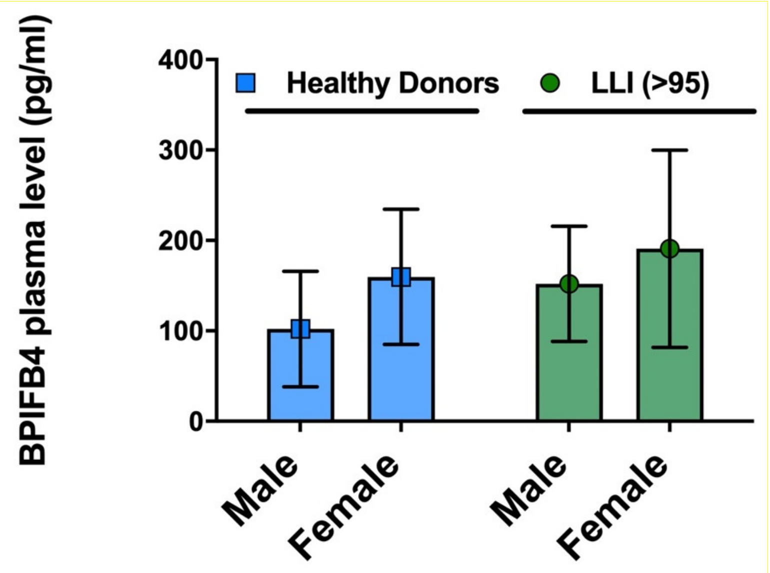

Supplementary Figure S1. ELISA quantification of BPIFB4 levels in plasma from both control volunteers (n = 20) and long-living individuals (LLIs, n = 51). No significant differences in BPIFB4 levels (pg/mL) were detected in n = 10 male with respect to n = 10 female healthy controls, neither in n = 15 males with respect to n = 36 female LLIs.
